# Clinical implementation of automated treatment planning for whole‐brain radiotherapy

**DOI:** 10.1002/acm2.13350

**Published:** 2021-07-10

**Authors:** Eun Young Han, Carlos E. Cardenas, Callistus Nguyen, Donald Hancock, Yao Xiao, Raymond Mumme, Laurence E. Court, Dong Joo Rhee, Tucker J. Netherton, Jing Li, Debra Nana Yeboa, Chenyang Wang, Tina M. Briere, Peter Balter, Mary K. Martel, Zhifei Wen

**Affiliations:** ^1^ Department of Radiation Physics University of Texas MD Anderson Cancer Center Houston TX USA; ^2^ Department of Radiation Oncology University of Alabama at Birmingham Birmingham AL USA; ^3^ Department of Radiation Oncology University of Texas MD Anderson Cancer Center Houston TX USA; ^4^ Department of Radiation Oncology Hoag Hospital Newport Beach CA USA

**Keywords:** automation, deep learning, whole brain

## Abstract

The purpose of the study was to develop and clinically deploy an automated, deep learning‐based approach to treatment planning for whole‐brain radiotherapy (WBRT). We collected CT images and radiotherapy treatment plans to automate a beam aperture definition from 520 patients who received WBRT. These patients were split into training (*n* = 312), cross‐validation (*n* = 104), and test (*n* = 104) sets which were used to train and evaluate a deep learning model. The DeepLabV3+ architecture was trained to automatically define the beam apertures on lateral‐opposed fields using digitally reconstructed radiographs (DRRs).

For the beam aperture evaluation, 1st quantitative analysis was completed using a test set before clinical deployment and 2nd quantitative analysis was conducted 90 days after clinical deployment. The mean surface distance and the Hausdorff distances were compared in the anterior‐inferior edge between the clinically used and the predicted fields. Clinically used plans and deep‐learning generated plans were evaluated by various dose–volume histogram metrics of brain, cribriform plate, and lens.

The 1st quantitative analysis showed that the average mean surface distance and Hausdorff distance were 7.1 mm (±3.8 mm) and 11.2 mm (±5.2 mm), respectively, in the anterior–inferior edge of the field. The retrospective dosimetric comparison showed that brain dose coverage (D99%, D95%, D1%) of the automatically generated plans was 29.7, 30.3, and 32.5 Gy, respectively, and the average dose of both lenses was up to 19.0% lower when compared to the clinically used plans. Following the clinical deployment, the 2nd quantitative analysis showed that the average mean surface distance and Hausdorff distance between the predicted and clinically used fields were 2.6 mm (±3.2 mm) and 4.5 mm (±5.6 mm), respectively.

In conclusion, the automated patient‐specific treatment planning solution for WBRT was implemented in our clinic. The predicted fields appeared consistent with clinically used fields and the predicted plans were dosimetrically comparable.

## 1 INTRODUCTION

Automation has been increasingly utilized in radiation therapy to improve standardization and efficiency. With the recent breakthrough in deep learning (DL), automation in clinical deployment can be further accelerated. Automated treatment planning in radiation therapy, including delineating targets with normal tissues and plan optimization, can greatly improve workflow and the quality of the treatment planning.^1^, ^2^, ^3^, ^4^

Conventional treatment planning is a time‐consuming task, which requires inputs from physicians, physicists, and dosimetrists. The most critical and time‐consuming task is to obtain the physician’s input for contours or field setup including MLC blocking.^5^, ^6^ That is why even a simple radiotherapy technique like whole‐brain radiation therapy (WBRT) can take up to several days from CT simulation to the start of treatment. In our clinic, the physician draws the MLC block on the lateral DRRs, then later reviews the manually generated WBRT plan and either accepts the plan or requests a field‐in‐field (FIF) technique to reduce hot spots (>107%) in the brain. Furthermore, some physicians prefer to apply a skin sparing technique which is shaping MLC to conform a patient’s cranium, to spare a patient’s hair and potentially decreases skin irritation in the posterior neck.^7^, ^8^

Schreibmann et al previously proposed an automated planning solution for whole‐brain radiotherapy.^5^ Their approach employed normal tissue auto‐contours that were matched to a database of WBRT patients allowing for automated field aperture definition through prior knowledge. A limitation of this approach is that anatomical similarity may not lead to appropriate field definition as these are generally dictated by the extension of metastasis. For example, individual patient’s disease may suggest extending the caudal border of the treatment fields from the caudal edge of C1 vertebrae to the caudal edge of C2 vertebrae.

**FIGURE 1:**
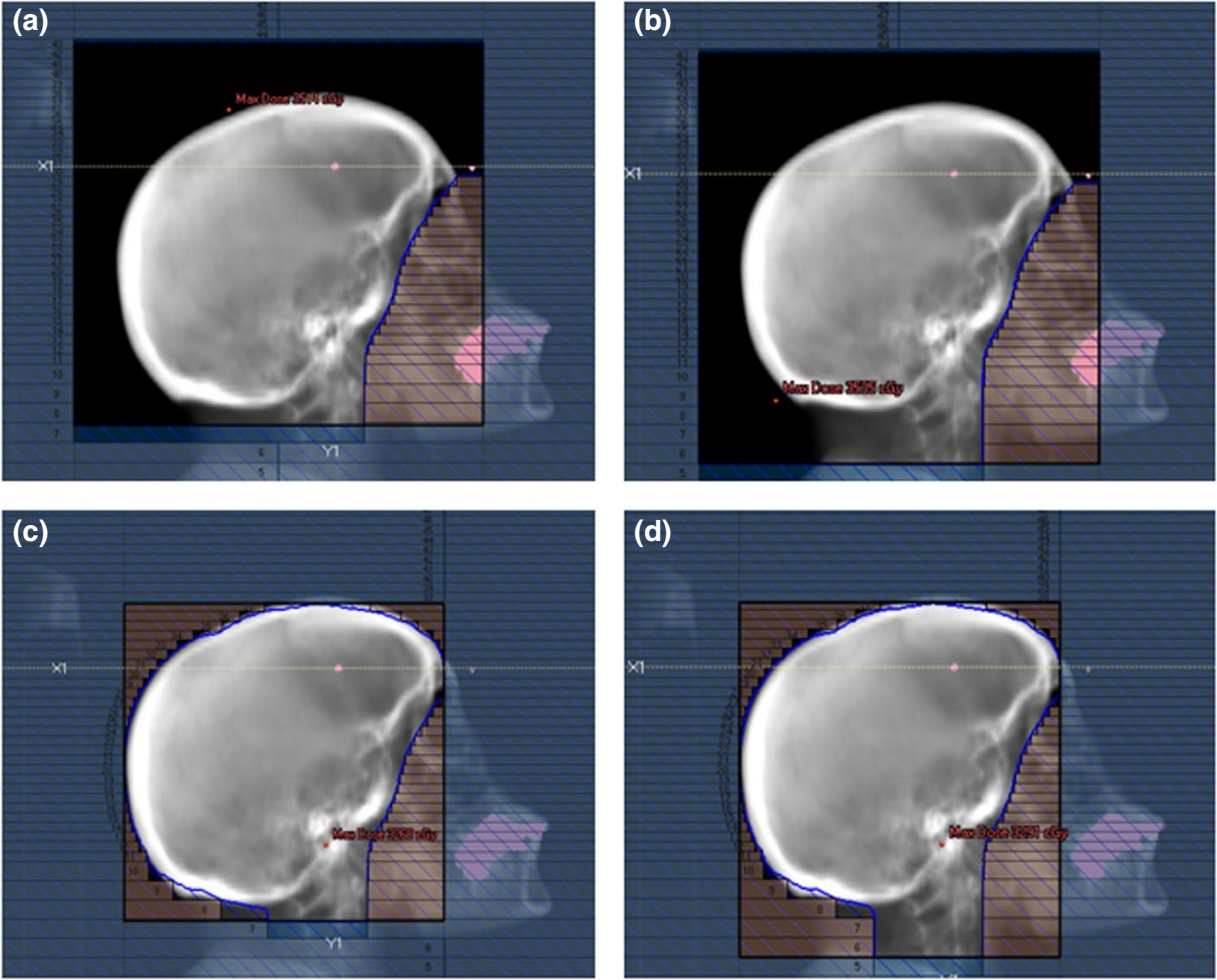
Example of field aperture options: (a) traditional whole‐brain radiotherapy (WBRT) with treatment extent to C1 vertebrae or (b) C2 vertebrae, and (c) scalp sparing WBRT to C1 vertebrae or (d) C2 vertebrae

**FIGURE 2:**
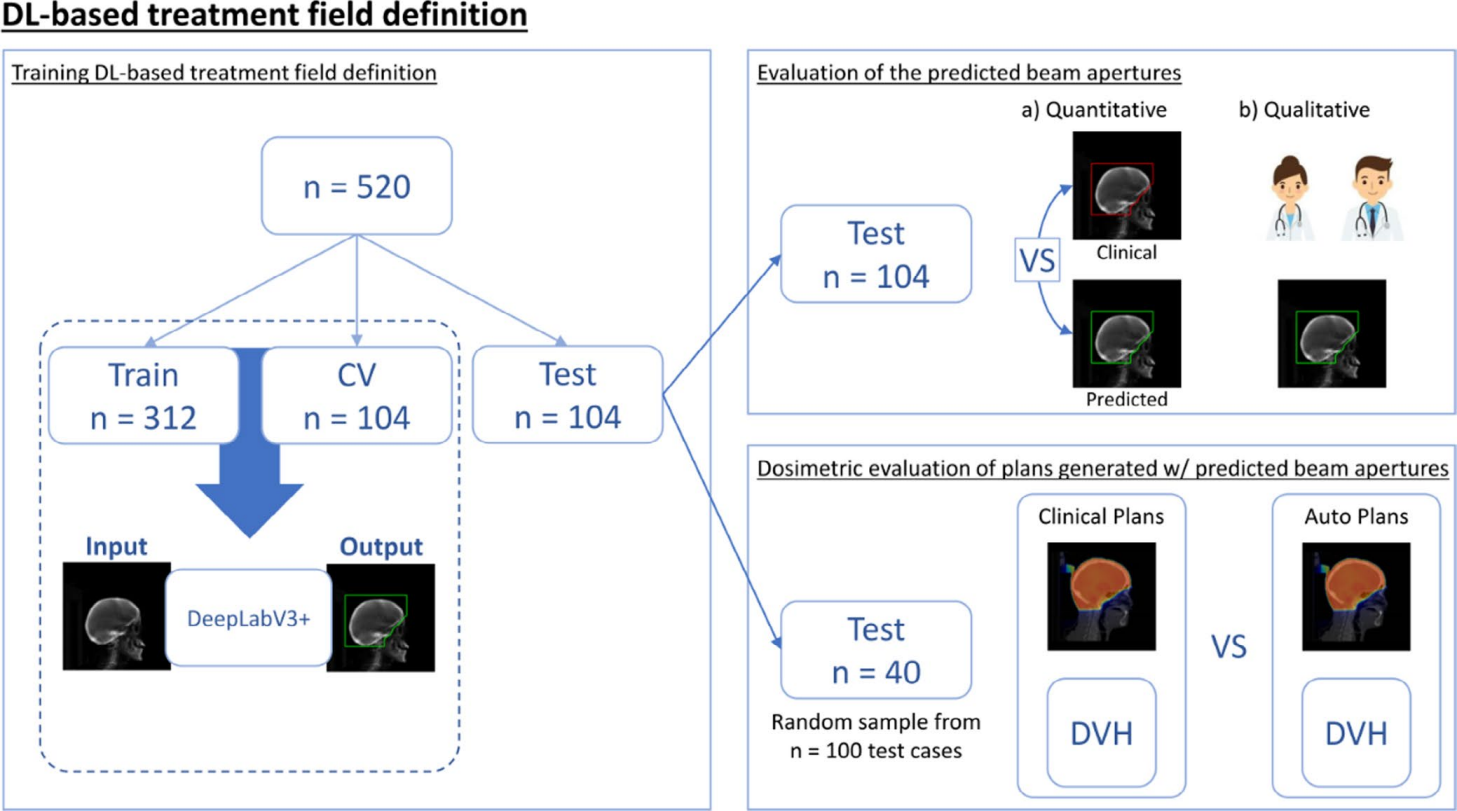
Schematic illustration of workflows including DL‐generated field definition and analysis

**FIGURE 3:**
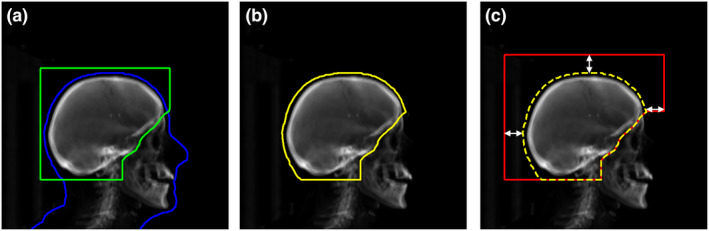
Schematic illustration of traditional WBRT field definition. (a) Digitally reconstructed radiograph (DRR) with clinically used beam aperture (green) and two‐dimensional projection of a patient's three‐dimensional body contour (blue). (b) Illustration of the inputs (DRR and contour) for deep learning‐based auto‐segmentation where the contour (yellow) is the intersection of the beam aperture and body contour projection. (c) Post‐processing step used to add skin flashing to the predicted mask (dashed yellow) to generate the final beam apertures (red). During testing, a value of 10 mm was chosen for skin flash for posterior, anterior, and cranial expansions (a value of 30 mm was used to highlight this step on this figure) based on clinical practice

To account for the patient‐specific field aperture definition, we proposed an automated treatment planning solution for WBRT which employs deep learning‐based field aperture definition providing a radiation oncologist with four field aperture options: (1) traditional WBRT with treatment extent to C1 vertebrae (Figure 1a), (2) traditional WBRT with treatment extent to C2 vertebrae (Figure 1b), (3) scalp sparing with treatment extent to C1 vertebrae (Figure 1c) and scalp sparing with treatment extent to C2 vertebrae (Figure 1d). In this study, we described the development of this tool, pre‐clinical evaluation, and post‐clinical evaluation.

## 2 MATERIALS AND METHODS

### 2.1 Deep learning‐based (DL) treatment field definition

#### 2.1.1 Model development

The planning CT scans and treatment plans of 520 WBRT cases previously treated at our institution were used to develop and evaluate an automatic field aperture definition model. This dataset was curated such that all cases were treated to cover the caudal extent of C1 or C2 vertebrae using a traditional WBRT field definition approach (Figure 1a,b). These cases were split into training (*n* = 312), cross‐validation (*n* = 104), and test (*n* = 104) sets. The DeepLabV3+ architecture^9^ was trained to automatically define the field apertures on laterally opposed digitally reconstructed radiographs (DRRs) from each patient’s CT scan using the physician‐drawn field apertures. The workflows for DL‐based field definition and analysis are shown in Figure 2. Note that the train and cross‐validation datasets are used during training; here, the model is trained on the training dataset and evaluated on the cross‐validation set during the training process, thus providing a real‐time measurement of the trained model’s performance on a separate (i.e., non‐training) dataset which allows for the optimization of the model’s hyper‐parameters. The final test set is then used to evaluate the final (i.e., best) model’s performance without introducing any training bias in the final analysis. DRRs were generated using in‐house software^10^ and the respective treatment plans (DICOM format) were used to generate image masks on the DRRs by extracting beam‐specific MLCs and jaw position information (Figure 3a). Due to the large variability found in skin flash (i.e., extent of the field outside of the patient), a pre‐processing step was used to train the deep learning model on the intersection of the body contour (projected on beams‐eye view [BEV]) and clinically used field aperture (Figure 3b). This pre‐processing step removed any noise due to variable skin flashing in training data. A post‐processing step was applied to add skin flash to the predicted masks (Figure 3c). This was accomplished by identifying the anterior/posterior/cranial extent of the predicted mask and applying a uniform flashing of 10 mm from the body contour to generate the final beam aperture prediction.

**FIGURE 4:**
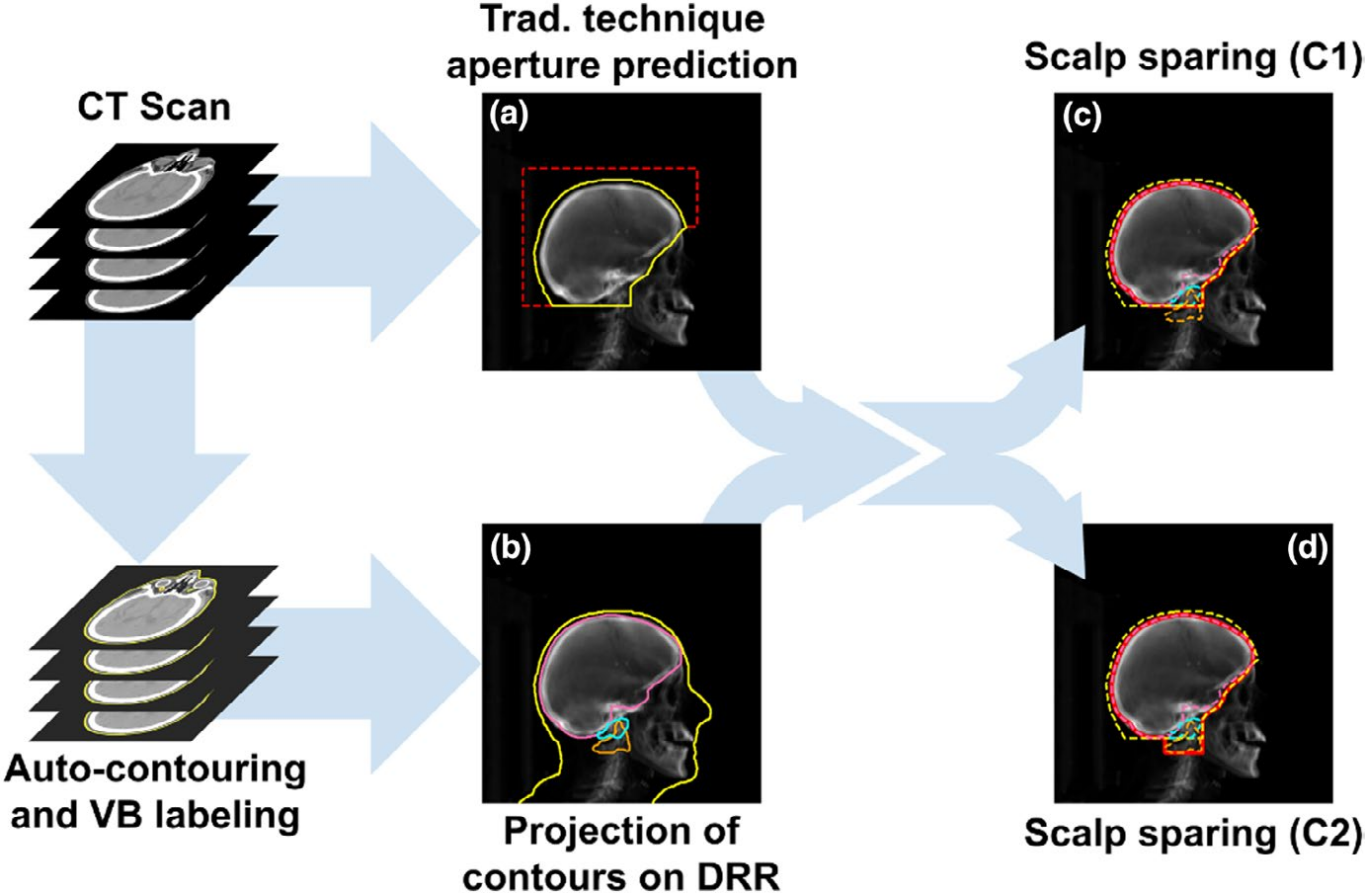
Schematic illustration of scalp sparing WBRT field definition. (a) Illustration of the inputs for deep learning‐based auto‐segmentation where the contour (yellow) is the intersection of the beam aperture and body contour projection (see Figure 3b). (b) Projection of contours of brain with 5 mm expansion and vertebras (VB) on DRR. (c) and (d) The caudal extension of the field to the caudal extent of C1 or the caudal edge of C2. The corresponding scalp sparing field apertures are shown by the solid red lines

Anterior/inferior edges of field apertures for scalp sparing WBRT were generated using the predicted masks (Figure 4a) from the trained deep learning model. Then, the brain and vertebral column are auto‐contoured on the CT scan using a deep learning model developed by Rhee et al.^11^ Prior to the projection of these contours to BEV, a uniform margin expansion of 5 mm is applied to the brain auto‐contour. The projected expansion of the brain contour is then used to define the superior/posterior edge of the scalp sparing field (Figure 4b), whereas the anterior/inferior edges are defined by the original traditional field predicted mask.

To provide the caudal extension of the field from the caudal extent of C1 to the caudal edge of C2 (Figure 4c,d), we used a deep learning algorithm to identify individual vertebral bodies^12^ within our vertebral column auto‐contours. This algorithm labels the centroids of each vertebral body. These centroids were used to approximate the extent of each vertebral column by calculating the mid‐point between centroids (i.e., mid‐point between C1 and C2 defines the caudal extent of C1).

#### 2.1.2 Evaluation of the predicted beam apertures

The WBRT fields (*n* = 104) in the test set were inspected by a radiation oncologist retrospectively. The focus of this evaluation was on verifying proper coverage in the anterior–inferior edges of the fields. Quantitative analysis was then conducted on the field border by comparing the predicted fields and clinically used fields using the mean surface distance (MSD) and the Hausdorff distance (HD).^13^ For the inferior field border (at the C1/C2 spine vertebrae interface), the distance between the prediction and clinical field edge was measured. For points along the curved anterior‐inferior field edge, the closest distance to the clinical field edge of each point was measured and the mean distance of all points was calculated. Only fields generated for the traditional field technique were evaluated. Historically, patients in our clinic were mostly treated using a traditional field definition technique; therefore, no available data to train a scalp‐sparing deep learning model. During the pre‐clinical evaluation of the automatic field aperture definition (using traditional technique only), some physicians expressed interest in having the “scalp sparing” technique available for clinical use; therefore, the decision was made to include the skin‐sparing option.

### 2.2 Automated plan generation

#### 2.2.1 Treatment planning system (TPS) integration

**FIGURE 5:**
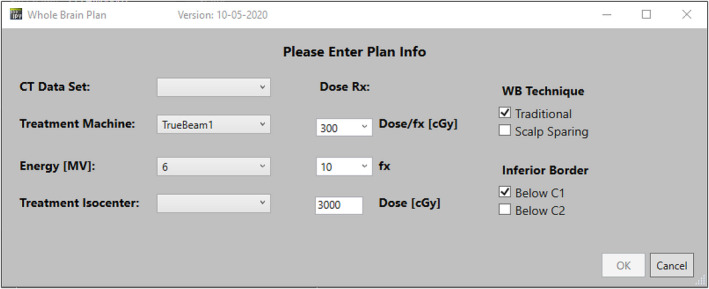
Whole‐brain plan interface. This window appears when the user launches the automated planning tool and is used to pre‐configure the automatically generated plans

**FIGURE 6:**
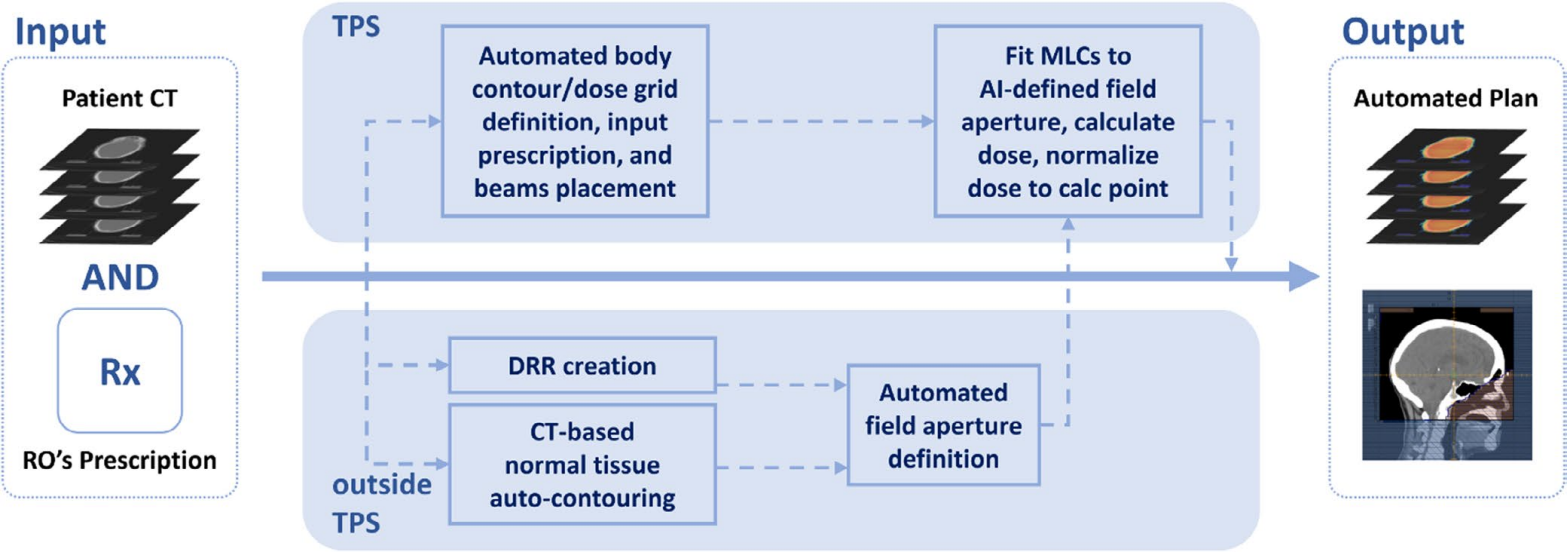
Schematic illustration of automatic planning process describing DL‐generated field definition integrated to treatment planning system

A graphical user interface (GUI) was created within RayStation (Figure 5, v9.0 RaySearch, Stockholm, Sweden) to automatically generate a WBRT plan using the TPS’s scripting. Within the GUI, the user is asked to input information about CT scan, treatment machine, energy, and to select the treatment isocenter. Furthermore, the user is asked to enter dose prescription/fractionation and patient‐specific treatment field technique (traditional vs. scalp sparing and C1 vs. C2) selected by the radiation oncologist. Once the selections are made, the automatic planning process begins as shown in Figure 6. First, external body contours and dose grid are automatically defined within the treatment planning system. Concurrently, a task is sent to a GPU‐enabled server to automatically generate normal tissue contours and automatically define field apertures using in‐house built deep learning algorithms (see Section 1.1). Normal tissue auto‐contours include brain, brainstem, eyes, lens, and spinal cord. Once these tasks are completed, the results are imported into the TPS and laterally opposed beams of equal weights (set at gantry angles of 270° and 90°) are created using the automatically defined field aperture. After the beams are generated, the dose is automatically calculated and normalized to the treatment isocenter (usually marked isocenter). This automatic planning process takes, on average, less than 5 min.

#### 2.2.2 Retrospective dosimetric comparison of clinical and deep learning‐generated plans

Next, 40 cases previously treated in our clinic were randomly selected for dosimetric comparison between clinical plans and deep learning‐generated plans (DL plan). Traditionally our physician does not specify a planning goal for WBRT in the planning directive other than a total dose and a number of fractions and rather visually review whether the brain dose coverage (D99%) would be 30 Gy to approve the plan. Plan quality indices were included for plan comparison ‐ Brain (D99% ‐ a dose covered by 99% of brain volume/D1%/D95%), cribriform plate (D99%), and lens average dose. The prescription dose was 30 Gy in 10 fractions and all plans were normalized 100% to the marked isocenter. Clinical plans were initially generated without a cribriform plate contour^14^ and later added for dosimetric comparison by two radiation oncologists. Two‐tailed paired‐samples t‐tests were conducted to evaluate the statistical significance of the differences between clinical plans and DL plans for the plan quality indices. The null hypothesis was no difference between the plans (*p* > 0.05).

#### 2.2.3 Clinical deployment

After 90 days of post‐clinical release, the evaluation of the DL plans was focused on the automatic definition of the beam apertures. The mean surface distance and Hausdorff distance were used to quantify the differences between the predicted and clinically used field edges focusing on the difference in the anterior–inferior edge of the fields.

## 3 RESULTS

### 3.1 Evaluation of WBRT automation tool

#### 3.1.1 1st evaluation of the predicted beam apertures before clinical deployment

All test cases (*n* = 104) were considered “clinically acceptable” by the radiation oncologist upon qualitative review of the predicted treatment fields. For the inferior field border, the average difference between DL predictions and clinical fields was 3.8 ± 3.0 mm. All predicted field apertures were correctly set to the junction of C1 and C2. Along the anterior–inferior field edge, the average (±standard deviation) mean surface distance and Hausdorff distance values were 7.1 mm (±3.8 mm) and 11.2 mm (±5.2 mm), respectively.

#### 3.1.2 Retrospective dosimetric comparison of clinical and deep learning‐generated plans

**FIGURE 7:**
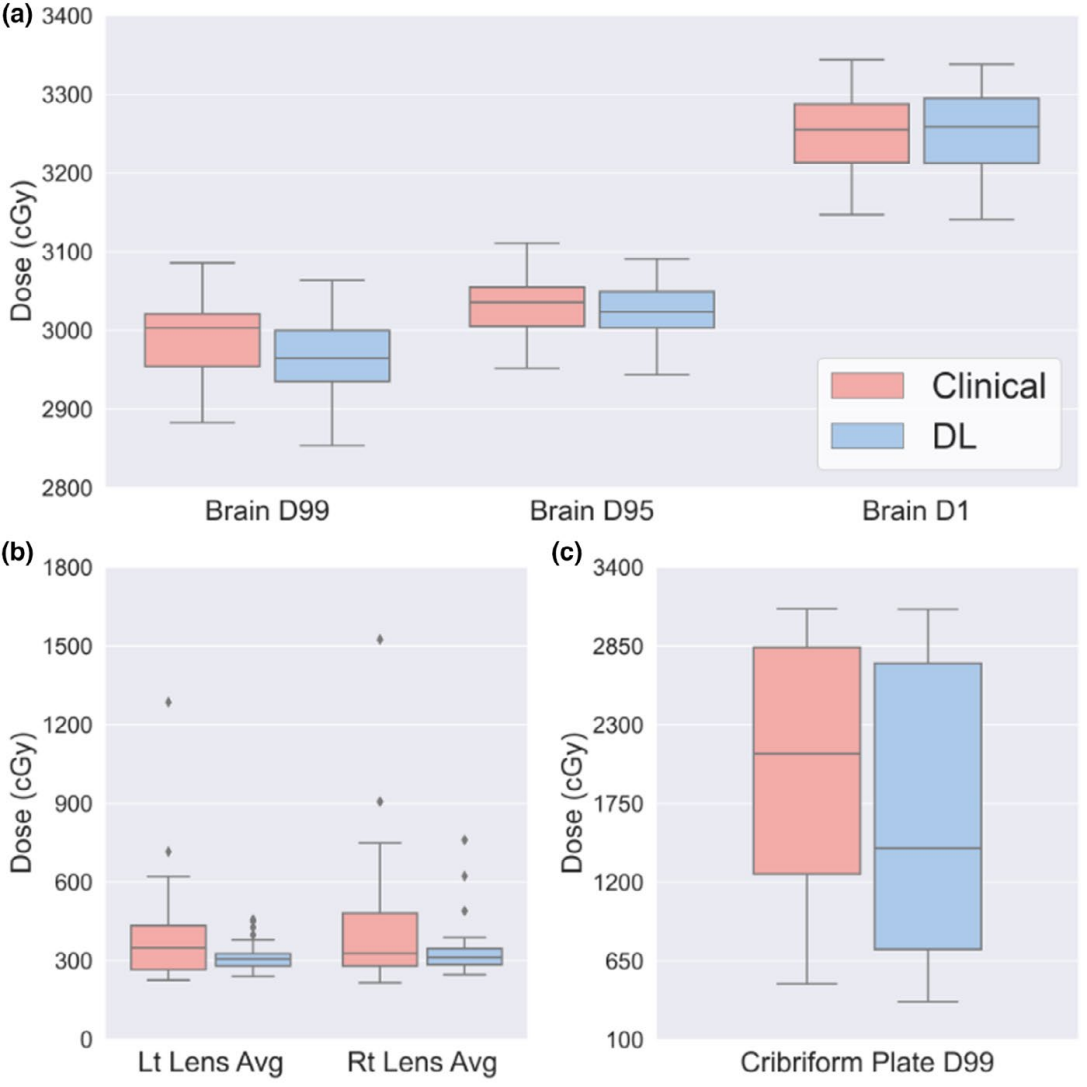
Box graph showing (a) brain dose coverage, (b) average dose of lenses, and (c) cribriform dose coverage from the clinical plan and DL generated plan (DL). The whiskers show the minimum and maximum values and the three horizontal lines illustrate each quartile (Q1 = 25th percentile, Q2 = 50th percentile, and Q3 = 75th percentile, respectively)

Table 1 shows the dosimetric comparison of clinical vs predicted dose (DL) metrics in averaged total dose ±standard deviation in cGy. As shown, brain dose coverage (D99%, D95%, D1%) of DL plans (*n* = 40) was 29.7 Gy ± 0.48 Gy, 30.3 Gy ± 0.34 Gy, and 32.5 Gy ± 0.52 Gy, respectively. Both methods met the implicit clinical goal of D99% of brain dose. The difference in D99% of brain (*p* = 0.003) was statistically significant but the difference in absolute dose was small (0.4 Gy). The difference in D95% (*p* = 0.53) and D1% (*p* = 0.63) was not statistically significant which means there is no significant difference between the two plans. The cribriform plate dose (D99%) was 15.9% (3.1 Gy) lower than clinical plans, but the difference was not statistically significant (*p* = 0.084). Since the cribriform plate was not traditionally defined during planning and the physician visually reviewed them, we were able to evaluate it only retrospectively after manually adding it to the plan. Last, the average dose of both lenses in the DL plan was lower by up to 19% than clinical plan (*p* < 0.05). Figure 7 shows box‐and‐whisker plots for brain, lens, and cribriform plate dose metrics.

**TABLE 1:** Dosimetric comparison of clinical vs. predicted average dose metrics (in cGy)

Brain D99%		Brain D95%		Brain D1%		Cribriform plate		LT Lens		RT Lens	
Clinical	DL	Clinical	DL	Clinical	DL	Clinical	DL	Clinical	DL	Clinical	DL
2991 ± 45	2967 ± 48	3031 ± 36	3026 ± 34	3251 ± 44	3254 ± 52	1977 ± 863	1662 ± 993	381 ± 186	313 ± 52	418 ± 241	334 ± 98

#### 3.1.3 2nd evaluation of the predicted beam apertures after clinical deployment

In the comparison of the anterior‐inferior border of the predicted and clinically used treatment fields, the average mean surface distance and Hausdorff distance between the predicted and clinically used fields were 2.6 mm (±3.2 mm) and 4.5 mm (±5.6 mm), respectively.

## 4 DISCUSSION

The DL‐based automated planning tool for WBRT has been clinically deployed at our institution. The automated WBRT planning tool can greatly improve the efficiency of clinical workflow and help to enhance treatment standardization to maintain a high standard in a busy radiation oncology clinic.

As Schreibmann et al reported,^5^ the presented study also shows comparable brain coverage and dose sparing in the lenses in the DL plans comparing to clinical plans. While their WBRT planning does not allow a patient‐specific plan customization, the presented study allows for a physician to select either traditional WBRT or scalp sparing WBRT with treatment extent to C1 or C2 vertebrae. Many studies have previously focused on automating individual tasks in the treatment planning process,^1^, ^2^, ^5^, ^15^, ^16^, ^17^, ^18^ yet very few offer end‐to‐end solutions which include automation of target definition, normal tissue contouring, beam selection, and dose optimization and calculation. Trained with clinical WBRT cases, the deep learning network could generate treatment fields comparable to clinical fields. Before clinical deployment, the predicted fields appeared consistent with the majority of training data within 7–11 mm. Automated WBRT plans are dosimetrically comparable to clinical plans regarding brain dose coverage and lower lens dose. After clinical deployment, a comparison of the anterior–inferior edge of the predicted and clinically used treatment fields showed that the predicted treatment fields are consistent within 3–5 mm with clinically used fields. There was a decrease in both distances (HD, MSD) in the post‐clinical analysis of field apertures; this might mean that the deep learning‐defined treatment fields produced clinically acceptable fields requiring only minor edits.

Since the WBRT plans with FIF have seen a steady increase in our clinical practice over the past decade (30% in 2012 to 61% in 2017), a future version of this tool will include an automatic FIF generation option to further improve dose homogeneity across the brain of WBRT patients and reduce the overall dosimetry workload and the analysis of clinical acceptance rate will be followed. In general, the use of the proposed tool would be deemed useful if it is used as a starting point on more than 50% of whole‐brain cases over the first 200 patients treated in our clinic after clinical release.

## 5 CONCLUSIONS

The WBRT automated treatment planning tool was implemented in our clinic. This tool provides a selection to account for patient‐specific field aperture definition. The predicted fields in the anterior–inferior edge appeared consistent with clinical data within 3–5 mm and automated plans are dosimetrically comparable to clinical plans with regard to brain dose coverage and lower lens dose.

## CONFLICT OF INTEREST

Nothing to disclose.

## AUTHOR CONTRIBUTION

Eun Young Han‐ writing manuscript, data analysis, Carlos E. Cardenas‐ writing manuscript, data analysis, model development, Callistus Nguyen – software development and clinical integration, Donald Hancock – software development and clinical integration, Yao Xiao – data analysis, manuscript review, Raymond Mumme – data analysis, manuscript review, Laurence E. Court – writing manuscript, manuscript review, Dong Joo Rhee – model development, manuscript review, Tucker J. Netherton – model development, manuscript review, Jing Li – data collection, manuscript review, Debra Nana Yeboa – data collection, manuscript review. Chenyang Wang, data collection, manuscript review. Tina M. Briere – data collection, manuscript review. Peter Balter – clinical integration, manuscript review, Mary K. Martel – clinical integration, manuscript review, Zhifei Wen – supervising research, manuscript review.
